# Evaluating the neuroprotective effects of progesterone receptors on experimental traumatic brain injury: The PI3K/Akt pathway

**DOI:** 10.1002/brb3.3244

**Published:** 2023-09-03

**Authors:** Ladan Amirkhosravi, Mohammad khaksari, Sedigheh Amiresmaili, Mojgan Sanjari, Parisa Khorasani, Morteza Hashemian

**Affiliations:** ^1^ Endocrinology and Metabolism Research Center Institute of Basic and Clinical Physiology Sciences Kerman University of Medical Sciences Kerman Iran; ^2^ Physiology Research Center Institute of Neuropharmacology Kerman University of Medical Sciences Kerman Iran; ^3^ Department of Physiology Bam University of Medical Sciences Bam Iran; ^4^ Department of Pathology, Pathology, and Stem Cells Research Center, Afzalipour Medical Faculty Kerman University of Medical Sciences Kerman Iran; ^5^ Neuroscience Research Center, Institute of Neuropharmacology Kerman University of Medical Sciences Kerman Iran

**Keywords:** neuroprotection, oxidative stress, PI3K/p‐Akt, progesterone receptor, pro‐inflammatory cytokines, traumatic brain injury

## Abstract

**Background:**

Studies have confirmed the salutary effects of progesterone (P4) on traumatic brain injury (TBI). This study investigated the beneficial effects of P4 via its receptors on TBI, and also whether progesterone receptors (PRs) can modulate TBI through PI3K/Akt pathway.

**Material and methods:**

Marmarou method was utilized to induce diffuse TBI in ovariectomized rats. P4 (1.7 mg/kg) or the vehicle (oil) was administered 30 min after TBI induction. Moreover, RU486 (PR antagonist) and its vehicle (DMSO) were injected before TBI induction and P4 injection. Brain Evans blue content, brain water content (WC), various oxidative stress parameters, IL‐1β levels, tumor necrosis factor‐α (TNF‐α), histopathological alterations, and also phosphorylated Akt (p‐Akt) and PI3K expressions in the brain were assessed 24 h after TBI. The veterinary comma scale (VCS) was measured before and after TBI at different times.

**Results:**

The findings revealed that P4 caused an increase in VCS and a decrease in brain WC, oxidative stress, TNF‐α and IL‐1β levels. RU486 inhibited the beneficial effects of P4 on these indices. Moreover, RU486 prevented the reduction of brain edema, inflammation, and apoptosis caused by P4. Moreover, P4 following TBI increased the expression of PI3K/p‐Akt protein in the brain. RU486 eliminated the effects of P4 on PI3K/p‐Akt expression.

**Conclusion:**

According to these findings, PRs are acting as critical mediators for the neuroprotective properties of P4 on oxidative stress, pro‐inflammatory cytokine levels, and neurological outcomes. PRs also play an important role in regulating the PI3K/p‐Akt expression and nongenomic function of P4.

## Graphical abstract

Studies have confirmed the salutary effects of progesterone (P4) on traumatic brain injury (TBI). This study investigated the beneficial effects of P4 via its receptors on TBI and whether progesterone receptors (PRs) can modulate TBI through PI3K/Akt pathway.

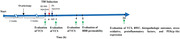

## 1 INTRODUCTION

One of the severe injuries that have long‐term consequences for the survivors is traumatic brain injury (TBI) (Faul et al., 2010). It also has a high mortality rate (Rodriguez et al., 2006). Local brain damage is caused by primary injury. Moreover, intracranial pressure spike caused by microvascular rupture, and axonal shearing contribute to secondary mechanisms that lead to an increase in Ca2^+^ channel opening, production of reactive oxygen species, cell death (Logsdon et al., 2015), and neuro‐inflammation in various brain regions including cortex, striatum, and hippocampus (Hernandez et al., 2018). Inflammation is caused by secondary injury by releasing immune mediators such as chemotactic factors and interleukins, brain edema, infiltration of peripheral blood cells, and blood–brain barrier (BBB) impairment (Thapa et al., 2021).

In the central nervous system (CNS), progesterone (P4) has neuroactive and neurosteroid functions, and its neuroprotective effects have been demonstrated in preclinical (Dong et al., 2018) and clinical studies (Mofid et al., 2016). P4 reduces brain edema (He et al., 2014) and retains BBB integrity (Si et al., 2014). It also decreases inflammatory response (Gutzeit et al., 2021) and pro‐inflammatory cytokines, including interleukin‐6 (IL‐6) (Sara et al., 2020), and transforming growth factor‐β (Khaksari et al., 2011; Sarkaki et al., 2011). Moreover, P4 suppresses oxidative stress and reduces cellular necrosis and apoptosis (Stanojlović et al., 2020).

Evidence indicates that a large part of protective effects of P4 after different types of injuries are associated with classical progesterone receptor (PR) (PR‐A and ‐B) activation (Petersen et al., 2013). PRs belong to the nuclear steroid hormone receptor super‐family of transcription factors regulating gene expression in a ligand dependent manner and intercede the so‐called genomic or classical effects of P4 both in female reproductive organs and in CNS (Schumacher et al., 2014). PRs are constitutively expressed in numerous brain areas and can begin gene transcription after specifically binding to P4 (Bali et al., 2012; Numan et al., 1999). These receptors regulate the expression of numerous genes required in target tissues’ development, differentiation, and proliferation (Obr & Edwards, 2012). PR also appears to be an essential receptor for controlling reactive gliosis and simultaneously producing pro‐inflammatory cytokines (Labombarda et al., 2015), characteristics of CNS traumas (Burda & Sofroniew, 2014). In this study, functional recovery and density of neurons were decreased, whereas neuro‐inflammation and total infarct volume were increased in mice with knockedout P4 receptors. Therefore, neuronal PR was found to be essential for reducing brain damage and motor function impairment following ischemia (Zhu et al., 2019). Mifepristone (RU486), a potent PR antagonist, reduces the neuroprotective effects of P4 (Ishihara et al., 2016; Lei et al., 2014). Pretreatment with mifepristone also reduces the neuroprotective effects of P4 on oxygen‐glucose deprivation/reoxygenation caused by death of neuronal cells (Ishihara et al., 2016).

Furthermore, both PR‐A and ‐B are located on the plasma membrane as well as inside the cell (Brinton et al., 2008). P4 activates its receptors’ membrane, which have a role in the adjustment of membrane/cytoplasmic signaling and the activation/phosphorylation of different kinases including extracellular signal‐regulated kinase (ERK), glycogen synthase kinase 3, and phosphoinositide 3‐kinase/protein kinase B (PI3K/Akt), which is also called “nongenomic mechanisms” (Guerra‐Araiza et al., 2007; Schumacher et al., 2014). Extranuclear signaling of PR and other steroid receptors may have an essential function in neurons (Schumacher et al., 2014). The PI3K/phosphorylated Akt (p‐Akt) is a key intermediary player in the signal transduction pathways that adjust cell survival, inflammation, metabolism, and cell growth (Zhao et al., 2006). Additionally, the PI3K/p‐Akt pathway has a neuroprotective role after ischemic injury (Wang et al., 2009). Treatment with P4 significantly increases p‐Akt after ischemic stroke (Ishrat et al., 2012), and this signaling pathway is involved in the PR‐dependent neuroprotection through P4 (Cai et al., 2012).

The neuroprotective and salutary effects of P4 after TBI, as well as effects of PI3K/p‐AKT in CNS injuries, have been reported in previous studies. This study aimed to assess the neuroprotective role of PR in mediating hormone effects by measuring oxidative stress biomarkers, pro‐inflammatory cytokines, BBB impairment, brain edema, neurological outcomes, and histopathological changes after TBI. This study also intended to survey the possible role of P4 receptors in regulating the protein expression of PI3K/p‐Akt as a nongenomic mechanism.

## 2 MATERIALS AND METHODS

### 2.1 Animal

Seventy‐two Wistar rats (female) (6–8‐week‐old) were taken from the Laboratory Animal Center of Kerman Medical University (220–250 g; Kerman, Iran). A standard situation (12 h light/dark cycle at 22 ± 2°C temperature) was provided for the animals and they had free access to food. The animals were kept and treated according to the standards. The Ethics Committee for Animal Care/Use at Kerman University of Medical Sciences approved all the experimental procedures (IR.KMU.REC.1401.148).

### 2.2 Ovariectomy operation

The rats were acclimatized to the condition of laboratory for 2 weeks. Mixed injection of 60/10 mg/kg ketamine/xylazine (i.p) (Alfasan) was used to anesthetize the rats. Then, a 1 cm incision was made in their sub abdominal area that had been shaved. After opening the skin, fascia, and abdominal muscles, fallopian tubes and vascular base of the ovaries were located at the proximal sections. They were then disconnected from the distal parts. Two milliliter of normal saline was used to wash abdominal cavity. A 4–0 silk surgical suture was utilized to close the incision. All the rats were ovariectomized (OVX) 2 weeks before the investigation in order to minimize or prevent intervention due to the estrus cycle (Azizian et al., 2018).

### 2.3 Drugs and animal groups

Six groups were considered, and OVX rats were randomly allocated in them (each group *n* = 12):

(a) Sham group: OVX rats were subjected to an incision of cranium skin after anesthesia, but brain injury was not induced.
(b) TBI group: OVX rats were subjected to brain injury after anesthesia.
(c) Oil group: OVX rats received an infusion of an equal volume of vehicle (sesame oil as P4 dissolvable) 30 min after TBI.
(d) P4 group: P4 (1.7 mg/kg) was injected to OVX rats 30 min after TBI (Soltani et al., 2016).
(e) Mifepristone (RU486) + P4 group: RU486 (5.0 mg/kg) was injected to OVX rats 1 and 1.5 h before TBI and P4 injection, respectively (Amirkhosravi, Khaksari, Sheibani, et al., 2021).
(f) VEH + P4 group: OVX rats received an equal volume of vehicle (DMSO as RU486 solvent) 1 and 1.5 h before TBI and P4 infusion, respectively.

**FIGURE 1:**
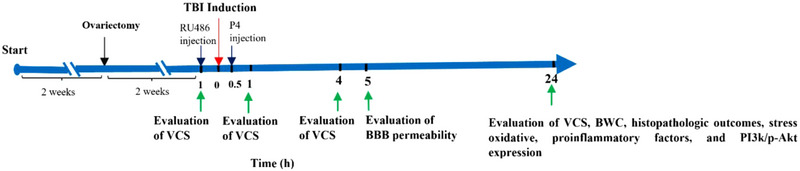
A simplified diagram of the study protocol and treatment schedule. BBB, blood brain barrier; BWC, brain water content; EB, Evans blue; P4, progesterone; PI3K, phosphatidylinositol 3 kinase; TBI, traumatic brain injury; VCS, veterinary comma scale.

Six rats in each group were used to determine BBB permeability, and other six rats were used to assess brain water content (BWC), stress oxidative, pro‐inflammatory factors, western blotting, and histopathological/neurobehavioral outcomes. The drugs were injected intraperitoneally. RU486 was prepared by Sigma and oil, and P4 was obtained from Aburaihan Pharmaceutical Company. Figure 1 depicts the experimental protocol schematic design.

### 2.4 Model of experimental TBI

A mix of ketamine–xylazine (50/5 mg/kg, i.p) was used to anesthetize the rats. They were then intubated using tracheal cannula to avoid hypoxia and control breathing. A heating pad was used to keep the animals at 37°C. To induce diffuse TBI by Marmarou method and impede skull fracture, a steel disk with 3 mm thickness and 10 mm diameter was bound to the skull bone by polyacrylamide glue along the crown of head between lambda and bregma points. The animals were located on a 10‐cm‐thick foam mattress lying on their stomachs, and from a height of 2 m, a 300‐g weight was dropped on their head. An animal breathing apparatus (TSE animal respirator) was instantly connected to the rats after trauma induction. The flow rate of TSE animal respirator was set at 2.5 L/min. The rats were removed from the pump and put in individual cages when they had recovered and started breathing on their own (Khaksari et al., 2013).

### 2.5 Assessment of brain water content (WC)

Twenty‐four hours after the brain injury, the wet/dry method was used to determine brain edema. After anesthesia, the brains were rapidly separated and equally divided. Then, the wet tissue (one hemisphere) was weighed and dried for 24 h at 100°C in an incubator. After drying, the tissue was weighed. Ultimately, the following formula was used to calculated BWC (Amirkhosravi, Khaksari, Soltani, et al., 2021):

[(wetweight−dryweight)/wetweight]×100%

### 2.6 Measuring BBB permeability

The BBB permeability was calculated using intravenous Evans blue (EB) dye 2% (20 mg/kg). Four hours after the trauma, the anesthetized animal (jugular vein) was injected with 20 mg/kg (1 mL/kg) of EB dye, and 1 h later its thorax was opened. After clamping the descending aorta, the intravascular dye was eliminated by injecting the solution of isotonic heparinized saline (approx. 300 mL) into the left ventricle. Saline was infused after cutting the jugular veins, until ejecting clear liquid. The researchers instantly separated, weighed, and homogenized the animal brain. Then, the homogenized brain was placed in 20 mL of solution [acetone solution (14 mL) + 1% sodium sulfate (6 mL)], and then it was shaken for 24 h. After that, 1 mL of trichloroacetic acid was combined with 1 mL of supernatant and left in a cold area for 3 min. The centrifugation was conducted on the solution at 2000 rpm for 10 min. Then, the supernatant EB dye absorption (1 mL) was quantified at the 620 wavelengths by a spectrophotometer (Pharmacia Biotech) (Soltani et al., 2015). The EB dye content was calculated by the following formula:

$$\mathrm{EB}\;\mathrm{dye}\left(\mu g\right)\mathrm{in}\;\mathrm{brain}\;\mathrm{tissue}\left(g\right)=13.24\times 20\times \mathrm{absorbance}/\mathrm{tissue}\;\mathrm{weight}.$$

### 2.7 Neurological outcome assessment

To assess the neurological outcomes, the veterinary coma scale (VCS) was utilized, and scores ranging from 3 to 15 were considered. The outcomes were divided into three categories, including eye function, motor function, and respiratory function. According to the VCS, higher scores indicate better neurological outcomes, whereas lower scores indicate a more severe neurological deficiency. Table 1 represents the scoring system. VCS was assessed 1 h before TBI and 1, 4, and 24 h after TBI by several trained researchers who were blind to the study groups (Amiresmaili et al., 2021).

**TABLE 1:** Veterinary coma scale.

Veterinary comma scale	Variable	Score
Motor function	Normal movement Mildly drowsy with spontaneous purposeful movements Lethargic, unable to stand, but maintains sternal recumbency Lethargic, withdraws to pinch, and lifts head with attention to visual stimuli; no sternal recumbency Withdraws or pedals to pinch Spontaneous pedaling Extensor posturing (spontaneous or to stimuli) Flaccid to stimuli	8 7 6 5 4 3 2 1
Eye function	Open Open on stimulation Normal eyelid reflexes No eyelid response to stimuli	4 3 2 1
Respiration	Normal Ataxic Apneic	3 2 1

### 2.8 Tissue homogenates provision

At the termination of treatment, brain tissue was removed and washed in 0.9% cold saline before being maintained at −70°C until utilization. For the preparation of homogenates, a homogenizer (Polytron PT 2100, KINEMATIC AG) was used. Homogenates were provided for tumor necrosis factor‐α (TNF‐α), IL‐1β, glutathione peroxidase (GPx), superoxide dismutase (SOD), nitric oxide (NO), and malondialdehyde (MDA) measurements according to the ratio of 100 mg tissue per 1 mL phosphate buffer (50 mmol/L; pH 7.5) containing 1 mm EDTA. The supernatants were utilized after centrifugation (20,000*g* for 10 min at 4°C) for biochemical analysis (Bandegi et al., 2014).

### 2.9 Measuring levels of malondialdehyde (MDA)

In this study, 0.5 mL of thiobarbituric acid and 1.5 mL of trichloroacetic acid were added to 125 μL of serum (thiobarbituric and trichloroacetic acids were obtained from Sigma Chemical Co.). A boiling water bath was used to submerge the test tubes for 45 min. The researcher added 1 mL of *n*‐butanol to the mixture and centrifugation was conducted at 750*g* for 10 min after cooling the compound. Finally, after stopping the pink phase, its absorbance was approximated at 534 nm. The tetra ethoxy propane standard curve was used to measure the MDA level (Rao et al., 1989).

### 2.10 Measurement of superoxide dismutase (SOD) and glutathione peroxidase (GPx)

The Randox kit (Cat No. SD125) was used 24 h after TBI to evaluate SOD and GPx activity in brain tissue based on the instructions of manufacturer.

### 2.11 Nitric oxide (NO) levels measurement

Plasma was first deproteinized using ZnSO4, 0.3 M NaOH. After that, the deprotonated plasma was blended to the Griess reagent (0.1% *N*‐(1‐naphthyl)ethylenediamine hydrochloride and 2% sulfanilamide in 5% phosphoric acid in deionized water) and vanadium(III) chloride. It was then incubated for 30 min, and its optical density was calculated (540 nm) (Yucel et al., 2012).

### 2.12 Evaluation of brain cytokines

Brain tissue IL‐1β and TNF‐α levels were measured using enzyme‐linked immunosorbent assay kits. The samples were examined on an automated enzyme‐linked immunosorbent assay plate reader (Model No. ELX‐80MS, Biotech). Data are being indicated as pg/mg of total protein.

### 2.13 Western blotting of PI3K/p‐Akt

The RIPA buffer comprised 0.1% Na‑deoxycholate, 1% NP‑40, 1 mM EDTA, 0.1% SDS, and 10 mM Tris–HCl (pH = 7.4). It also included protease inhibitors (10 μg/mL of aprotinin, 1 mM sodium orthovanadate, 2.5 μg/mL of leupeptin, and 1 mM phenylmethylsulfonyl fluoride). The brain tissue was homogenized in this buffer, and then, centrifugation was conducted at 14,000 rpm and 4°C for 15 min. The total cell fraction was obtained from the final supernatant. In the Bradford method, the protein standard is bovine serum albumin (BSA), and concentrations of protein were assessed by this method (Bio Rad Laboratories). The 9% SDS–PAGE gel was used to load identical protein volumes. After that, they were transmitted to PVDF membranes. Blocking of membranes was conducted in Tris‐buffered saline by 5% nonfat dried milk with Tween 20 using blocking buffer at room temperature for 3 h (150 mM NaCl, pH 7.5, 20 mM Tris–HCl, 0.1% Tween 20). Then, phospho (p)‐Akt (Ser473) (Cell Signaling Technology Inc. 1:1000) and PI3K protein (p85) (Cell Signaling Technology, Inc. 1:1000) were investigated by primary antibodies at 4°C overnight. Membranes washing was performed in TBST buffer (5 min, 3 times), and a secondary antibody Goat‐anti‐rabbit‐IgG (H + L)‐HRP (Bio‐Rad Laboratories 1:10,000) was utilized for incubation at room temperature for 90 min (1:15,000, GE Healthcare BioSciences Corp.). Antibody–antigen complexes were tracked by ECL system with exposure to Lumi‐Film chemiluminescent detection film (Roche). For expression intensity analysis, lab analysis software (UVP) was used. β‐Actin antibody (antibody from cytomatin gene company, 1:1000) was utilized to control loading (Yu et al., 2007).

### 2.14 Brain histopathology

Histopathological outcomes were evaluated 24 h after TBI in terms of changes in edema, vascular congestion, inflammation, apoptosis, and microglia proliferation. The brain tissue was fixed in 10% paraformaldehyde, and then, an automatic microtome (LEICA) was used to prepare 4‐μm sections before being stained by and hematoxylin–eosin. Two pathologists, who were blinded to the drug used and animal groups, assessed the histopathological changes using an optical microscope. The scores ranging from 3 = severe, 2 = moderate, 1 = mild, and 0 = low were used for apoptosis, edema, inflammation, and vascular congestion. The amount of damage under the microscope with 10% magnification was graded as follows: severe (apoptosis was observed in all high‐power field), moderate (apoptosis was observed in more than one high‐power field), mild (apoptosis was observed in a high‐power field), and low (apoptosis was rarely observed in a high‐power field). The isolated parenchymal cells and extracellular spaces were taken into account for brain edema. The edema was classified under a microscope with a magnification of 10× as follows: severe (edema above 50%), moderate (edema between 10% and 50%), and mild (edema less than 10%). The existence of inflammation was evidenced by the presence of eosinophils, plasma cells, lymphocytes, and polymorphonuclear leukocytes. The microglia proliferation was described as +1, +2, +3, and +4 (Shamsi Meymandi et al., 2018).

### 2.15 Data analysis

The data have been displayed as mean ± SEM. Shapiro–Wilk was utilized to check the data normality. One‐way ANOVA was used to compare the variables of BBB permeability, WC of brain, Il‐1β and TNF‐α, oxidants and antioxidants, expression of PI3k/p‐Akt, and histopathological variables between the study groups, followed by the Tukey‐post hoc test. Furthermore, for post hoc analysis, the Tukey test was conducted. Moreover, to examine the neurological outcomes (VCS) before and 1, 4, and 24 h after TBI between the study groups, we used the repeated measure test and Bonferroni's post hoc test. A *p*‐value of less than .05 was considered statistically significant.

## 3 RESULTS

### 3.1 P4 effect and its antagonist on brain edema

Table 2 shows variations in BWC 24 h after TBI. The TBI increased BWC compared to the sham (*p* < .001). P4 reduced BWC in comparison with the oil and TBI groups (*p* < .01). Moreover, RU486 elevated this parameter relative to the VEH + P4 (*p* < .05).

**TABLE 2:** Comparison of brain water content (BWC) (%) in different experimental groups.

Parameter	Sham	TBI	Oil	P4	VEH + P4	RU + P4
**BWC**	75.85 ± 0.45	81.15 ± 0.5^***^	79.16 ± 0.23^***^	77.16 ± 0.33^#^	77.6 ± 0.25	79.11 ± 0.61^†^

*Note*: Values are mean ± SEM. *n* = 6 rats/group.

Abbreviations: P4, progesterone; TBI, traumatic brain injury; VEH, vehicle.

^***^ *p* < .001 versus sham.

^#^ *p* < .05 versus oil.

^†^ *p* < .05 versus VEH + P4.

### 3.2 P4 effects and its antagonist on BBB permeability

Variations in brain EB content are represented in Table 3. Relative to the sham group, TBI increased brain EB content (*p* < .001). P4 decreased this index relative to the oil and TBI groups (*p* < .01). Moreover, RU486 raised this parameter in comparison with the VEH + P4 group (*p* < .001).

**TABLE 3:** Comparison of brain Evans blue (EB) content in different experimental groups.

Parameter	Sham	TBI	Oil	P4	VEH + P4	RU + P4
**Brain EB content**	9 ± 0.44	33.43 ± 0.51^***^	31.12 ± 0.62^***^	18.08 ± 0.17^###^	19.92 ± 0.35	26.11 ± 0.62^†††^

*Note*: Values are mean ± SEM. *n* = 6 rats/group.

Abbreviations: P2, 17β‐estradiol; RU, RU486; TBI, traumatic brain injury.

***p < .001 versus sham.

^###^ *p* < .001 versus oil.

^+++^ *p* < .001 versus VEH + P4.

### 3.3 P4 effect and its antagonist on neurologic outcomes

**FIGURE 2:**
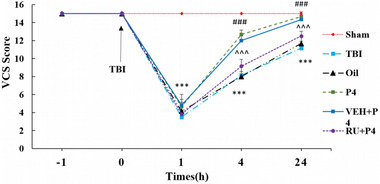
Relationship between veterinary coma scale (VCS) and traumatic brain injury (TBI) at different times before (−1 h) and after (1, 4, and 24 h) TBI in the different groups (*n* = 6 in each group). Values are mean ± SEM. ***p < .001 versus sham, ###*p* < .001 versus oil, †††*p* < .001 versus vehicle (VEH) + progesterone (P4). RU, RU486.

Figure 2 reveals neurological score (VCS) variations in the study groups at various times of post TBI. Significant differences were not seen in neurologic scores between the groups before the TBI induction. Compared to the sham group, the TBI decreased the neurological score at 24, 4, and 1 h after the TBI (*p* < .001). P4 increased VCS relative to the oil and TBI groups at 24 and 4 h (*p* < .001). RU486 at 24 and 4 h lessened this index relative to the VEH + P4 (*p* < .001).

### 3.4 P4 effects and its antagonist on antioxidant activity

**FIGURE 3:**
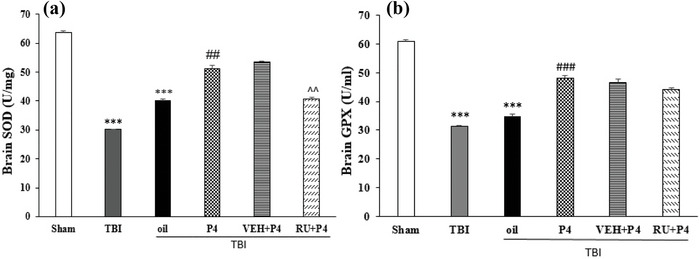
Comparison of superoxide dismutase (SOD) (μ/mg) and glutathione peroxidase (GPx) (μ/mg) after traumatic brain injury (TBI) in the different groups (*n* = 6 in each group). Values are mean ± SEM. (a) ***p < .001 versus sham, ##*p* < .01 versus oil, ^^*p* < .01 versus vehicle (VEH) + progesterone (P4). (b) ***p < .001 versus sham, ##*p* < .01 versus oil. RU, RU486.

Figure 3 contrasts brain GPx and SOD levels in various study groups. Compared to the sham group at 24 h after TBI, the TBI reduced brain GPx and SOD levels (*p* < .001) and led to a decrease in antioxidants activity. In comparison to the oil and TBI groups, P4 increased SOD activity (*p* < .01) Figure (3a) and caused an antioxidant activity. RU486 reduced brain SOD activity compared with the VEH + P4 (*p* < .001) and inhibited the antioxidant activity of P4. Brain GPx level significantly increased in the P4 group compared to the TBI and oil groups and also increased antioxidant activity (*p* < .001) (Figure 3b).

### 3.5 Effects of P4 and its antagonist on brain MDA and NO level

**FIGURE 4:**
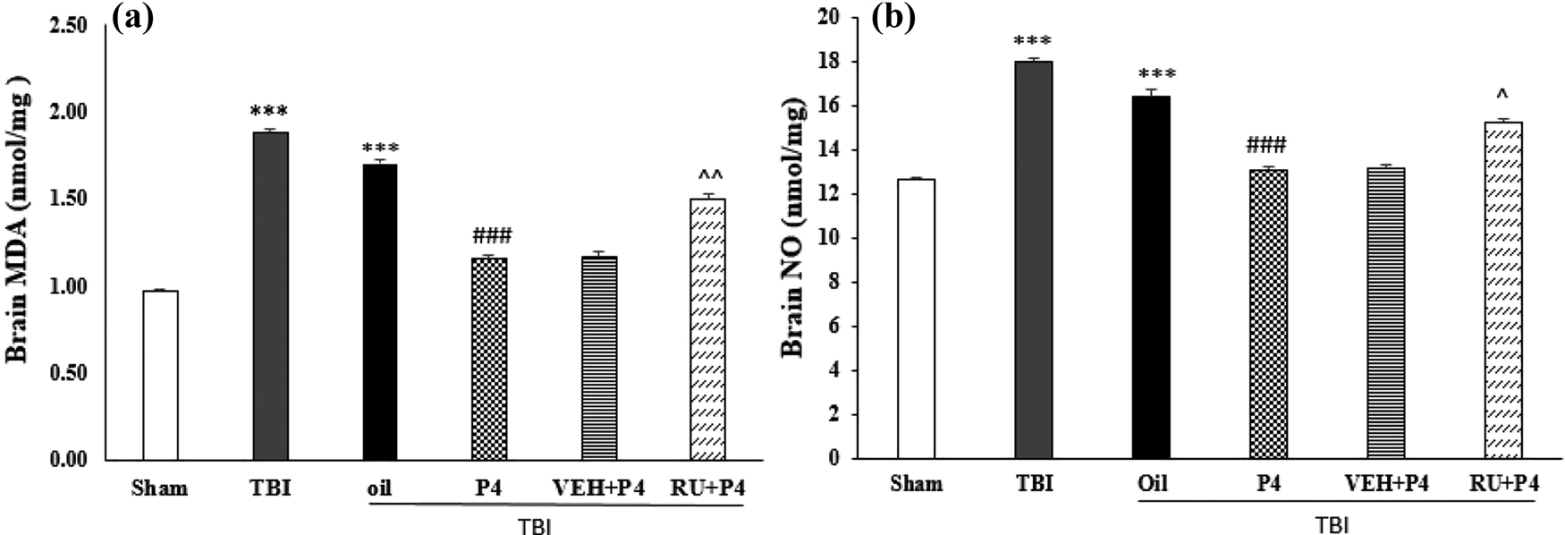
Comparison of brain malondialdehyde (MDA) (nanomol/mg) and nitric oxide (NO) (μmol/mg) after traumatic brain injury (TBI) in the different groups (*n* = 6 in each group). Values are mean ± SEM. (a) ***p < .001 versus sham, ###*p* < .001 versus oil, ^^*p* < .01 versus vehicle (VEH) + progesterone (P4). (b) ***p < .001 versus sham, ###*p* < .001 versus oil, ^*p* < .05 versus VEH + P4. RU, RU486.

Brain MDA and NO levels were compared in different study groups, which are displayed in Figure 4. TBI raised brain MDA and NO levels, which was higher in the sham (*p* < .001), and also increased oxidants activities. Compared to the oil group, the brain MDA level was decreased by P4 (*p* < .001), which also inhibited oxidant activity (Figure 4a). Moreover, Figure 4a shows that, compared to VEH + P4 group, brain MDA activity increased in the RU486 + P4 group (*p* < .01) followed by increased oxidant activity. Figure 4b shows that NO level in the P4 group was lower than the oil group (*p* < .001), leading to a decrease in oxidant activities. In comparison, RU486 enhanced brain NO level relative to the VEH + P4 group, and also oxidant activities (*p* < .001).

### 3.6 Effects of P4 and its antagonist on the IL‐1β and TNF‐α level of the brain

**FIGURE 5:**
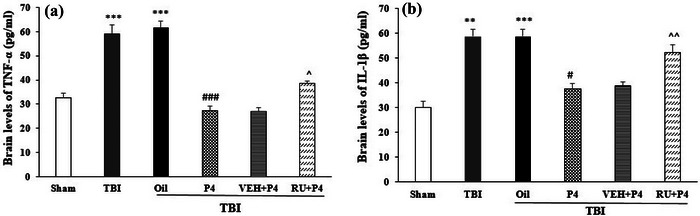
Comparison of brain tumor necrosis factor‐α (TNF‐α) and IL‐1β (pg/mg) level after traumatic brain injury (TBI) in the different groups (*n* = 6 in each group). Values are mean ± SEM. (a) ***p < .001 versus sham, ###*p* < .001 versus oil, ^*p* < .05 versus vehicle (VEH) + progesterone (P4). (b) ***p < .001 versus sham, #*p* < .05 versus oil, ^^*p* < .01 versus VEH + P4. RU, RU486.

Figure 5 compares different groups in terms of the mean TNF‐α and IL‐1β levels in the brain after the TBI. TBI raised TNF‐α and IL‐1β levels, which were higher in the sham group (*p* < .001) (Figure 5a, b) and also indicated an increase in neuro‐inflammation. Compared with the oil group, P4 treatment reduced TNF‐α (*p* < .001) and IL‐1β (*p* < .05) levels and led to a reduction in inflammation. Furthermore, RU486 elevated the TNF‐α (*p* < .05) and IL‐1β (*p* < .01) levels relative to the VEH + P4 group, indicating that this antagonist suppresses the anti‐inflammatory effect of P4 (Figure 5a,b).

### 3.7 Effects of P4 and its antagonist on PI3K and p‐Akt protein expression level

**FIGURE 6:**
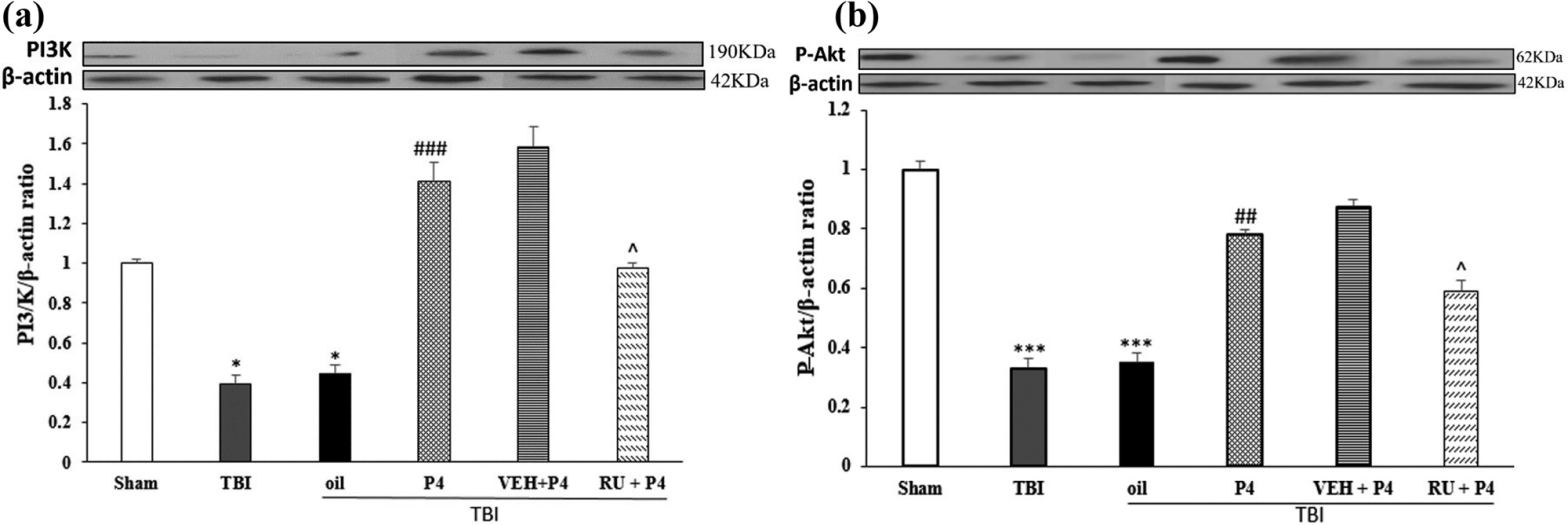
The effect of progesterone (P4) on PI3K and phosphorylated Akt (p‐Akt) levels at 24 h post‐traumatic brain injury (TBI). (a) *p < .05 versus sham, ###*p* < .001 versus oil, ^*p* < .05 versus vehicle (VEH) + P4. (b) ***p < .001 versus sham, ##*p* < .01 versus oil, ^*p* < .05 versus VEH + P4 (see complete unedited blots in supplemental material). RU, RU486.

Figure 6a compares different studied groups in terms of the level of PI3K expression in the brain after the TBI. In comparison with the sham group, PI3k protein expression level significantly decreased in the oil and TBI groups (*p* < .001). P4 treatment increased the level of PI3k expression, which was higher in the oil group (*p* < .01). Compared to the VEH + P4 group, RU486 prevented the effect of P4 on the protein expression level (*p* < .05). Figure 6b shows brain p‐Akt expression level after TBI in the studied groups. Compared to the sham group, the activated Akt level (p‐Akt) decreased in the oil and TBI groups (*p* < .001). P4 reduced the p‐Akt level after trauma (*p* < .01). Moreover, RU486 inhibited the P4 effect on expression of this protein relative to the VEH + P4 group (*p* < .05) (see complete unedited blots in Supporting Information section).

### 3.8 Histopathological findings

**FIGURE 7:**
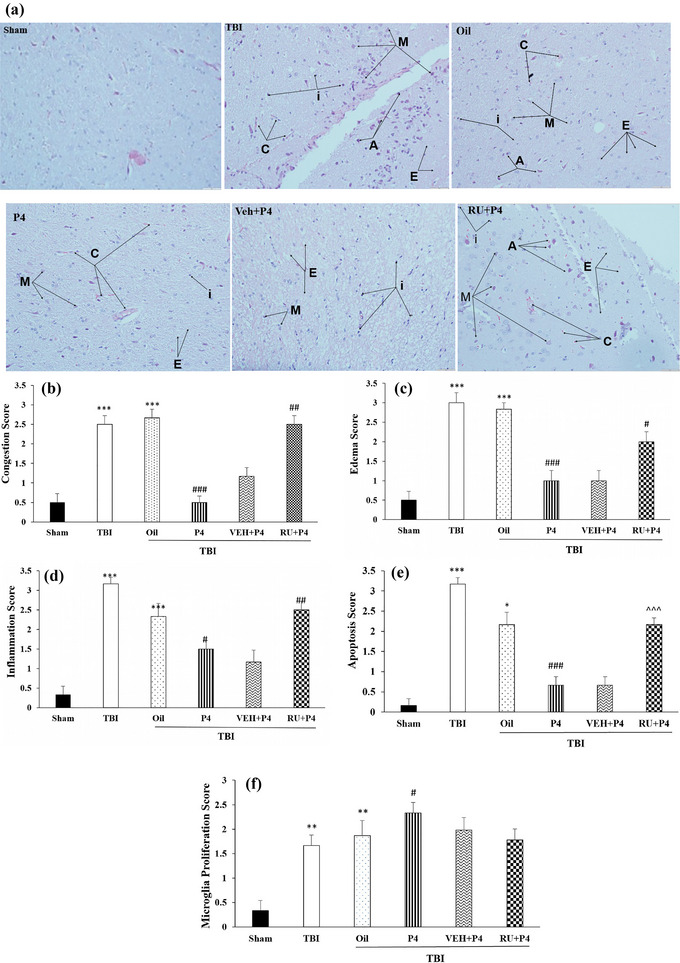
Histopathological findings (a–f): (a) these figures (hematoxylin & eosin, 200×) showed that progesterone receptors might decrease congestion, edema, inflammation, apoptosis, and microglia proliferation in brain tissue. C, E, i, A, and M arrows, respectively, showed congestion, edema, inflammation, apoptosis, and microglia proliferation; (b) comparison of congestion score: ***p < .001 versus sham, ###*p* < .001 versus oil, ##*p* < .01 versus vehicle (VEH) + progesterone (P4). (c) Edema score: ***p < .001 versus sham, ###*p* < .001 versus oil, and #*p* < .05 versus VEH + P4; (d) inflammation score: ***p < .001 versus sham, #*p* < .05 versus oil, ##*p* < .01 versus VEH + P4; (e) apoptosis score: ***p < .001 and *p < .05 versus sham, ###*p* < .001 versus vehicle, ^^^*p* < .05 versus VEH + P4; (f) microglia proliferation score: **p < .01 versus sham, #*p* < .01 versus oil. *n* = 6 rats/group. Values are mean ± SEM. RU, RU486; TBI, traumatic brain injury.

The histopathological parameters were assessed, including edema, congestion, inflammation, apoptosis, and microglia proliferation in the brain tissue following TBI. Histopathological images in experimental groups are represented in Figure 7a. At 24 h after injury, congestion score raised in the TBI and oil group compared to the sham group (*p* < .001). Compared to the oil group, this variable was lower in P4 group (*p* < .001). Meanwhile, compared to the VEH + P4 group, a significant increase was seen in this parameter in the RU + P4 group (*p* < .01) (Figure 7b).

Moreover, at 24 h after injury, edema score raised in TBI and oil groups in comparison with the sham group (*p* < .001). Similar to congestion, in P4 treatment group, this score was lower than oil group (*p* < .001). Moreover, RU486 prevented this inhibitory effect of P4, so compared to the vehicle group, this index was higher in RU + P4 group (*p* < .05) (Figure 7c).

The results of inflammation score in the study groups at 24 h after injury are presented in Figure 7d. Compared to the sham group, a significant increase was observed in this variable in the oil and TBI groups (*p* < .001). P4 treatment reduced this inflammatory score compared to the oil group (*p* < .05). Moreover, inflammation in the RU + P4 group increased in comparison with the VEH + P4 group (*p* < .001).

Our data disclosed that the apoptotic score was greater in TBI (*p* < .001) and oil (*p* < .05) groups in comparison with the sham group (Figure 7e). Compared to the oil group, P4 decreased the apoptotic score (*p* < .001). In line with the inflammation score, RU486 increased this index in comparison with the VEH + P4 group (*p* < .001) (Figure 7e).

Figure 7f represents that microglia proliferation score raised in oil and TBI groups compared to the sham group (*p* < .001). Administration of P4 increased microglia proliferation (*p* < .05), meanwhile RU486 could not inhibit the P4 effect.

## 4 DISCUSSION

P4 is considered to have various neuroprotective effects in neuronal injury models, including TBI (Sayeed & Stein, 2009; Zhang et al., 2017). The current study attempted to determine the P4 receptor's role in mediating the P4 neuroprotective effects on oxidative stress, levels of pro‐inflammatory cytokines, neurological outcomes, and regulating PI3K/p‐Akt expression following TBI. We showed that: (1) Pretreatment with RU486 reversed the efficacy of P4 on brain edema, BBB permeability, and neurological outcome; (2) after TBI, P4 decreased oxidants (MDA, NO) and increased antioxidants (GPx, SOD) levels, and the receptor antagonist reversed this effect; (3) and P4 treatment attenuated TBI‐stimulated IL‐1β and TNF‐α levels, and P4 effects were inhibited by RU486; (4) P4 also increased the expression of PI3K and p‐Akt, and RU486 had an inhibitory effect on PI3K/p‐Akt expression after TBI. Moreover, P4 prevented the changes in histopathological parameters of brain damage (such as congestion, edema, inflammation, and apoptosis), and RU486 inhibited these effects.

Our results revealed that the P4 injection in OVX rats caused a reduction in BBB permeability and brain edema and also improved neurologic scores after trauma. Consistent with this study, it has been reported that treatment with exogenous P4 protects the brains of male and female rodents against ischemic damage (Gibson et al., 2011; Yu et al., 2017). P4 improves neurological function, restores the leaked BBB, and reduces BWC and inflammatory response (Yu et al., 2017). The multiple neuroprotective mechanisms of P4 in neuronal injury include (1) reducing brain edema (Soltani et al., 2016), excitotoxicity (Smith, 1991), apoptosis, and myelin repair (Djebaili et al., 2004); (2) impeding the expression of COX‐2, IL‐6, NF‐κB (Cutler et al., 2007), and TNF‐α (Si et al., 2014); (3) making changes in the expression of AQP‐4 (Soltani et al., 2016); (4) reducing the levels of matrix metalloproteinases (MMPs) (Wang et al., 2013); (5) lipid peroxidation (Roof et al., 1997); (6) increasing vessel density and occluding expression (Yu et al., 2017). Moreover, P4 increases the oxidants’ activity against oxidative stress induced by ischemic injury (Aggarwal et al., 2010; Roof et al., 1997).

The results of the present study indicated that the administration of RU486 omitted the P4 effects on BBB permeability, brain edema, oxidative stress, and pro‐inflammatory factors. A lack of increase in the neurologic scores was also detected after the use of P4. The neuroprotective effects of P4 could not be fully ruled out, despite the significant differences that were observed between the groups of RU + P4 and P4. In this regard, it can be said that, there is a probability that at least parts of salutary effects of P4 are not dependent only on the P4 receptors. The independent effects of P4 might be due to its membrane receptors rather than its classic receptors or its intrinsic antioxidant attributes (Coronel et al., 2014). Although P4 receptors interact with membrane‐associated kinases outside the nucleus, they are ligand‐activated transcription factors (Grimm et al., 2016; Zhu et al., 2017). These receptors are also expressed throughout the brain, including hypothalamic nuclei, cerebral cortex, and subcortical structures (Gofflot et al., 2007). RU486, as a pure P4 antagonist (Ishihara et al., 2016), competes for binding with P4 nuclear and membrane receptors (A and B) and also reverses the effects of P4. This antagonist exerts its effects through one of the following mechanisms: increasing the degradation of PR (Azeez et al., 2021), competing for binding with P4 receptor ligand, limiting PR nuclei dimerization and localization, downregulating the receptors, restricting transcriptions (Brinton et al., 2008), and reducing available receptors. The neuroprotective effects of P4 have been confirmed on neurons and neuro‐inflammatory responses via PR. This is in line with our results regarding the role of PRs (Zhu et al., 2019). Moreover, infarct volumes and neurological deficits increase with the elimination of brain PR (Zhu et al., 2017). Moreover, classic P4 receptors mediate neural viability, expression of AQP‐4 (Zhu et al., 2019), and expression of brain derived neurotrophic factor (Jodhka et al., 2009). P4 also has beneficial effects on ischemia‐induced neuronal death (Ishihara et al., 2016) and neuro‐inflammation (Lei et al., 2014). Jodhka et al. (2009) showed that these effects are sensitive to RU486. In line with this study, it has been demonstrated that RU486 inhibits P4 effects on TNF‐α, and COX‐2 expression in lipopolysaccharide‐stimulated BV‐2 microglia (Lei et al., 2014). P4 receptors have also been shown to improve oxidative factors in neuropathic diabetics (Mokhtari et al., 2022). The anti‐edema effects are also associated with PR. In other brain injuries such as meningitis cerebral edema, P4 anti‐edema effect is antagonized by RU486 (Benzel & Gelder, 1988).

Numerous studies have suggested that the PI3K/AKT pathway activation has a crucial neuroprotective role in TBI by regulating autophagy and anti‐apoptosis responses (Miao et al., 2016; Wei et al., 2017). In addition, research has shown a role of PI3K/Akt signaling in oxidative stress suppression induced by intracerebral hemorrhage (Hannan et al., 2020). This study also revealed that P4 eliminated the decrease in expression of PI3K and p‐Akt after TBI, and RU486 had an inhibitory effect on PI3K/p‐Akt expression induced by P4. The effect of RU486 on PI3K/p‐Akt expression indicates that the activity of nongenomic signaling pathway can be affected by PRs. Studies have reported that P4 enhances the phosphorylation of Akt/PKB (Koulen et al., 2008; Singh, 2001) in the hippocampus and cerebellum (Guerra‐Araiza et al., 2009). Downstream effectors of PI3K signaling, such as Akt/PKB, are used to repress apoptotic cell death and impede the Bcl2 protein (Noshita et al., 2001). In addition, PI3K/Akt signaling pathway can be used by P4 to decrease inflammation and protect brain (Ishrat et al., 2012). Regarding the specific PI3K inhibitor “LY294002,” evidence has shown that ischemia‐induced brain damage can be prevented, and for that, P4 neuroprotection PI3K signaling is required (Cai et al., 2012). In a P4R‐dependent manner, P4 causes the immediate and temporary activation of PI3K‐Akt pathway (Migliaccio et al., 1998; Vallejo et al., 2005). It has been shown that a membrane‐associated PR (mPR) without a functional DNA‐binding domain will cause this steroid to have a nongenomic effect (Guerra‐Araiza et al., 2009). Evidence has demonstrated that the Src‐ERK signaling pathway is activated by P4R (Boonyaratanakornkit et al., 2008) by the phosphorylation of ERK1/2 (Cai et al., 2008). The membrane‐impermeant P4‐BSA, which interacts with the intracellular PR, elicits ERK phosphorylation (Cai et al., 2012; Jodhka et al., 2009). It has also been revealed that the neuroprotection effect mediated by PI3K is not dependent on P4R. Given that RU486 inhibits the classic P4 receptor, the mediation of P4 neuroprotective effect is exerted by classic PR or maybe PR membrane in a complementary manner.

Primary outcomes of histopathological parameters were surveyed in this study, including changes in edema, congestion, apoptosis, inflammation, and microglia proliferation after TBI, and the findings showed that P4 prevented these changes. P4 receptors mediated these beneficial effects because RU486 inhibited all these effects except for microglia proliferation. In this study, RU486 could not inhibit P4 effect on microglia proliferation. Probably, P4 induced its effect through other PRs, such as mPR (Meffre et al., 2013). Studies have shown that P4 inhibits the secretions of IL‐1β, TNF‐α, and NO synthetic enzyme and also eliminates macrophage infiltration, which is in line with the current study (Medhi et al., 2010; Sarkaki et al., 2011). In addition, it has been reported that P4 can alleviate inflammatory reaction by inhibiting NF‐κB (Li et al., 2015), and COX‐2 (Hardy et al., 2008) expression through its receptors (PR‐A and PR‐B) (Kobayashi et al., 2010).

Apoptosis is a programmed and initiative brain cellular death (Li et al., 2015). It has been shown that P4 causes a significant change in the apoptotic cell death markers (p‐Bad, caspase‐3, Bcl‐2, and TUNEL staining) probably through p‐Akt signaling pathway, which is consistent with the present study. Besides, RU486 causes a rise in caspase‐3 activity and DNA fragmentation (Svensson et al., 2001). Additionally, RU486 removes P4's neuroprotective properties in ischemia‐induced neuronal death (Cai et al., 2012). Not assessing fluorescent staining for microglia proliferation markers and apoptosis is one of the limitations of this study. Therefore, it is recommended to use this method in future studies. We suggest future studies to investigate: (1) the nongenomic pathway by P4 conjugated to BSA, (2) the role of this pathway in other effects of P4 (i.e., behavior outcome), and (3) the PI3K/Akt protein expression level in the specific area of the brain.

In general, the results of present study indicated that classic P4 receptors had interceding mediating effect after TBI. The blockade of these receptors by RU486 (PR antagonist) after TBI abolished the neuroprotective effects of P4, including edema inhibition, decrease of BBB permeability, and improvement of neurological outcomes, oxidative stress, and inflammation. Additionally, we demonstrated that RU486 decreased the expression of PI3K/p‐Akt protein. Therefore, classical PRs play a critical role in regulating PI3K/p‐Akt expression and nongenomic neuroprotective effects of P4 following TBI.

## AUTHOR CONTRIBUTIONS

**Ladan Amirkhosravi**: Formal analysis; investigation; writing—original draft; writing—review and editing. **Mohammad Khaksari**: Conceptualization; methodology; funding acquisition; supervision. **Sedigheh Amiresmaili**: writing—review and editing. **Mojgan Sangari**: Methodology. **Parisa Khorasani**: Methodology. **Morteza Hashemian**: Editing draft and Methodology.

## CONFLICT OF INTEREST STATEMENT

No conflict of interest is declared in this study.

### PEER REVIEW

The peer review history for this article is available at https://publons.com/publon/10.1002/brb3.3244.

## Supporting information

Supporting Information

## Data Availability

The data that support the findings of this study are available on request from the corresponding author.
