# Supplementary material for: Evaluating the neuroprotective effects of progesterone receptors on experimental traumatic brain injury: The PI3K/Akt pathway
Source: Brain Behav. 2023 Sep 3;13(11):e3244. doi: 10.1002/brb3.3244 (PMC10636406; doi:10.1002/brb3.3244)
Supplement: Supplementary file 1 — Supporting Information [file BRB3-13-e3244-s001.docx]

**The determination of GPx activity**

Glutathione peroxidase (GPx) assay was determined according to the method described by Paglia and Valentine (1967) by Randox kit (UK; Cat NO.SD125) (1). The Randox GPx assay Kit measures GPx activity indirectly by a coupled reaction with glutathione reductase. Glutathione reductase is an enzyme, which is responsible for regenerating the reduced form of oxidized glutathione (GSSG) which is generated when GPx3 produces an organic peroxide. Absorbance decreases at 340 nm (A340) when NADPH oxidizes to NADP^+^, which is a spectrophotometric means for measuring the activity of GPx enzyme.

**The determination of SOD activity**

The total Superoxide dismutase (SOD) activity was determined according to the protocol of Randox kit (UK; Cat NO.RS504). (SOD) functions as a catalyst in the dismutation of the superoxide radical (O^-^_2_) into hydrogen peroxide (H_2_O_2_) and elemental oxygen (O_2_). In the Randox assay kit, superoxide ions (O^-^_2_), that are produced either by xanthine oxidase (XOD) or through the conversion of xanthine to uric acid and hydrogen peroxide, are responsible for converting NBT to NBT-diformazan. NBT-diformazan absorbs light at 560 nm. SOD lowers the speed of NBT-diformazan formation by reducing the concentration of superoxide ion. SOD activity is measured by calculating the extent of reduction that happens in the presence of NBT-diformazan in an experimental sample.

**Enzyme-linked immunosorbent assay for quantitative detection of Rat TNF-α (Hangzhou, Eastbiopharm, China)**

**Component of kit:**

TNF-α antibody pre-coated plates

Standards 1-4: Out of kit and in the -20°C

Biotin-conjugated anti-TNF-α antibody, 1 vial 5500 µL

HRP-Avidin, 1 vial 5500 µL

HRP, 1 vial 180 µL

Substrate, 1 vial 5500 µL

Stopping, 1 vial 3 mL

10X washing buffer, 1 vial 40 mL

**Other materials needed**

Pipettes and pipettors

96-well ELISA plate reader

Distilled water (DW)

**Samples**

The antibodies used in this kit are able to recognize Rat TNF-α in the serum, tissues, cell culture supernatant, only, so, this kit is not suitable for detection of TNF-α in other races, such as mouse or human.

**Standards**

The standards are ready to use and are out of kit at -20 ºC. Accordingly, standards number 4, 3, 2, and 1 contains 200, 100, 50, and 0 pg/mL. Standard 4 leads to OD between 1.3 to 1.6, standard 3 leads to OD between 0.6 to 0.9, standard 2 leads to OD between 0.3 to 0.5, and standard 0 leads to OD between 0.05 to 0.08.

**Kit sensitivity**

The kit can determine up to 2 pg/mL and its intra-assay is 3-4% and inter-assay is 8-10%.

**Preparation of solutions**

The washing buffer is in 10X format, so add 360 mL DW to make 1X washing buffer. For preparation of HRP-Avidin, please add whole the HRP vial components to the HRP-Avidin. The component is ready to use in the procedure of the kit. The HRP-AVIDIN prepared is stable for 1 week. In the case of using the kit in the several times, please prepare the HRP-Avidin for one strip (8 vials), as follows: 458 µL of HRP-Avidin with 15 µL of HRP.

**Preparation of samples**

In the case of evaluation of TNF-α in the tissues and supernatant of the cell cultures, please make 8 series serial dilution of the samples and the dilution with OD: 1.5 needs to be considered for all the samples. The tissues need to be homogenized in the RIPA buffer containing anti-proteases.

**Experimental procedure**

Add 50 µL/well of standard number 4 to A1, standard number 3 to B1, standard number 2 to C1, and standard number 1 to D1. Accordingly, add 50 µL of your (pre-diluted) samples to other wells and incubate the plate 1 hour in the room temperature and on the shaker (180 RPM). All the incubations are at room temperature. Aspirate wells and wash 3 times with 250 µL/well 1X wash buffer. Allowing time for soaking (~1 minute) during each wash step increases the effectiveness of the washes. Blot plate on absorbent paper to remove any residual buffer.

Add 50 µL/well biotin-conjugated anti-TNF-α antibody (Detection Ab) to all wells.

Seal the plate and incubate at room temperature for 1 hour.

Wash the wells as described in the second step.

Add 50 µL/well HRP-Avidin to all wells.

Seal the plate and incubate at room temperature for 30 minutes.

Wash the wells 5 times as described in the second step.

Add 50 µL/well substrate to all wells and incubate for 15 minutes on the shaker (180 RPM).

Add 25 µL/well stopping to all wells.

Read plates at 450 nm. If wavelength subtraction is available, subtract the values of 570 nm from those of 450 nm and analyze data.

**Enzyme-linked immunosorbent assay for quantitative detection of Rat IL-1ß** (Hangzhou, Eastbiopharm, China)

**Component of kit:**

IL-1ß antibody pre-coated plates

Standards 1-4: Out of kit and in the -20°C

Biotin-conjugated anti-IL-1ß antibody (CN: KPG-RIL1ßD), 1 vial 5500 µL

HRP-Avidin, 1 vial 5500 µL

HRP, 1 vial 180 µL

Substrate, 1 vial 5500 µL

Stopping, 1 vial 3 mL

10X washing buffer, 1 vial 40 mL

**Other materials needed.**

Pipettes and pipettors

96-well ELISA plate reader

Distilled water (DW)

**Samples**

The antibodies used in this kit are able to recognize Rat IL-1ß in the serum, tissues, cell culture supernatant, only, so, this kit is not suitable for detection of IL-1ß in other races, such as human or mouse.

**Standards**

The standards are ready to use and are out of kit at -20 ºC. Accordingly, standards number 4, 3, 2, and 1 contains 200, 100, 50, and 0 pg/mL. Standard 4 leads to OD between 1.3 to 1.6, standard 3 leads to OD between 0.6 to 0.9, standard 2 leads to OD between 0.3 to 0.5, and standard 0 leads to OD between 0.05 to 0.08.

**Kit sensitivity**

The kit can determine up to 1 pg/mL and its intra-assay is 3-4% and inter-assay is 8-10%.

**Preparation of samples**

In the case of evaluation of IL-1ß in the tissues and supernatant of the cell cultures, please make 8 series serial dilution of the samples and the dilution with OD: 1.5 needs to be considered for all the samples. The tissues need to be homogenized in the RIPA buffer containing anti-proteases.

**Experimental procedure**

Add 50 µL/well of standard number 4 to A1, standard number 3 to ß1, standard number 2 to C1, and standard number 1 to D1. Accordingly, add 50 μL of your (pre-diluted) samples to other wells and incubate the plate 1 hour in the room temperature and on the shaker (180 RPM). All the incubations need to be at room temperature.

Aspirate wells and wash 3 times with 250 µL/well 1X wash buffer. Allowing time for soaking (~1 minute) during each wash step increases the effectiveness of the washes. Blot plate on absorbent paper to remove any residual buffer.

Add 50 µL/well biotin-conjugated anti-IL-1ß antibody (Detection Ab) to all wells.

Seal the plate and incubate at room temperature for 1 hour.

Wash the wells as described in the second step.

Add 50 µL/well HRP-Avidin to all wells.

Seal the plate and incubate at room temperature for 30 minutes.

Wash the wells 5 times as described in the second step.

Add 50 µL/well substrate to all wells and incubate for 15 minutes on the shaker (180 RPM).

Add 25 µL/well stopping to all wells.

Read plates at 450 nm. If wavelength subtraction is available, subtract the values of 570 nm from those of 450 nm and analyze data.

**Histopathological tissue preparation**

The brain tissue sample was washed with a 0.9% saline solution and fixed in 10% paraformaldehyde. A coronal section of the brain was prepared. After that, the slices prepared in 10% formalin were placed one and a half hours in 70% alcohol, one and a half hours in 80% alcohol, and one and a half hours in 90% alcohol. Then, the brain sections were placed twice, one and a half hours in 100% alcohol. Then, it was put twice in xylene for an hour and a half. It was put in melted paraffin at a temperature of 65°C for two and a half hours, all the tissues were embedded in paraffin, and 4-micron-thick sections were taken by automatic microtome (LEICA, Germany). The prepared sections were stained with hematoxylin and eosin.

**The actual whole blots**

To maintain blinding throughout the study, numbering was used, and each represents the following:

**1: sham 2: TBI 5: oil 6: P4 7: G1 8: VEH+P4 9: ICI+E2 10: G15+E2 11: G15+E2-BSA 13: RU+P4 12: ICI+G15+E2-BSA 3: E2-BSA 4: E2**


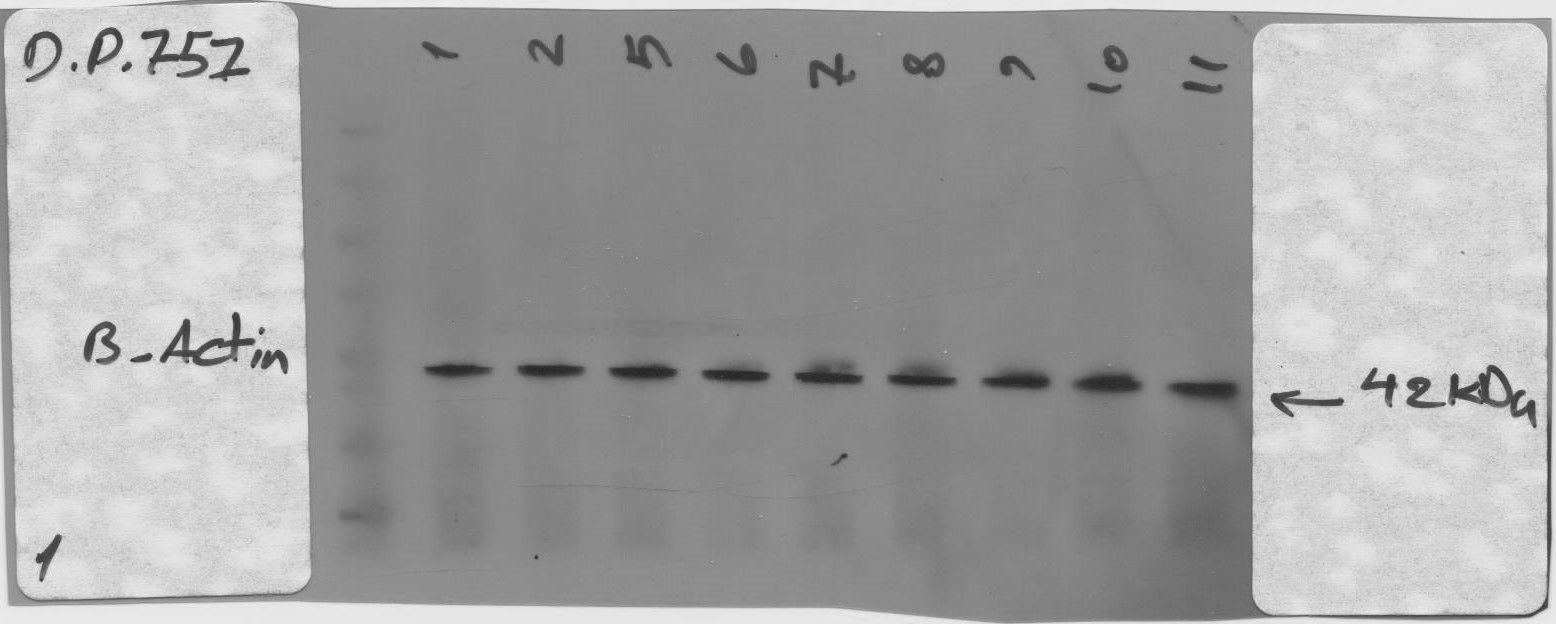


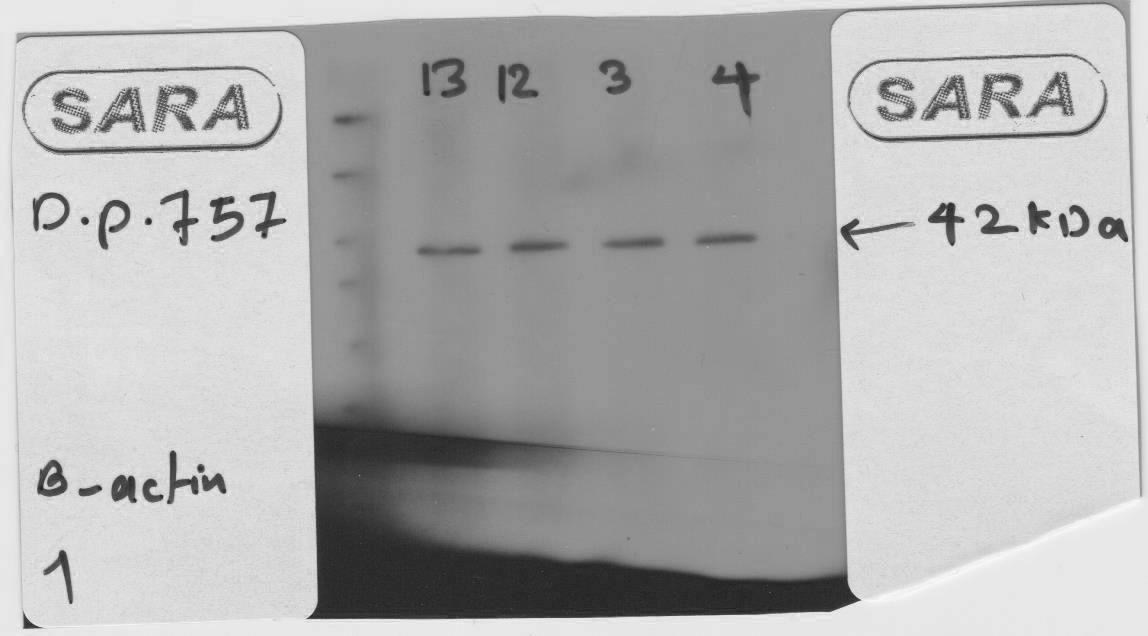


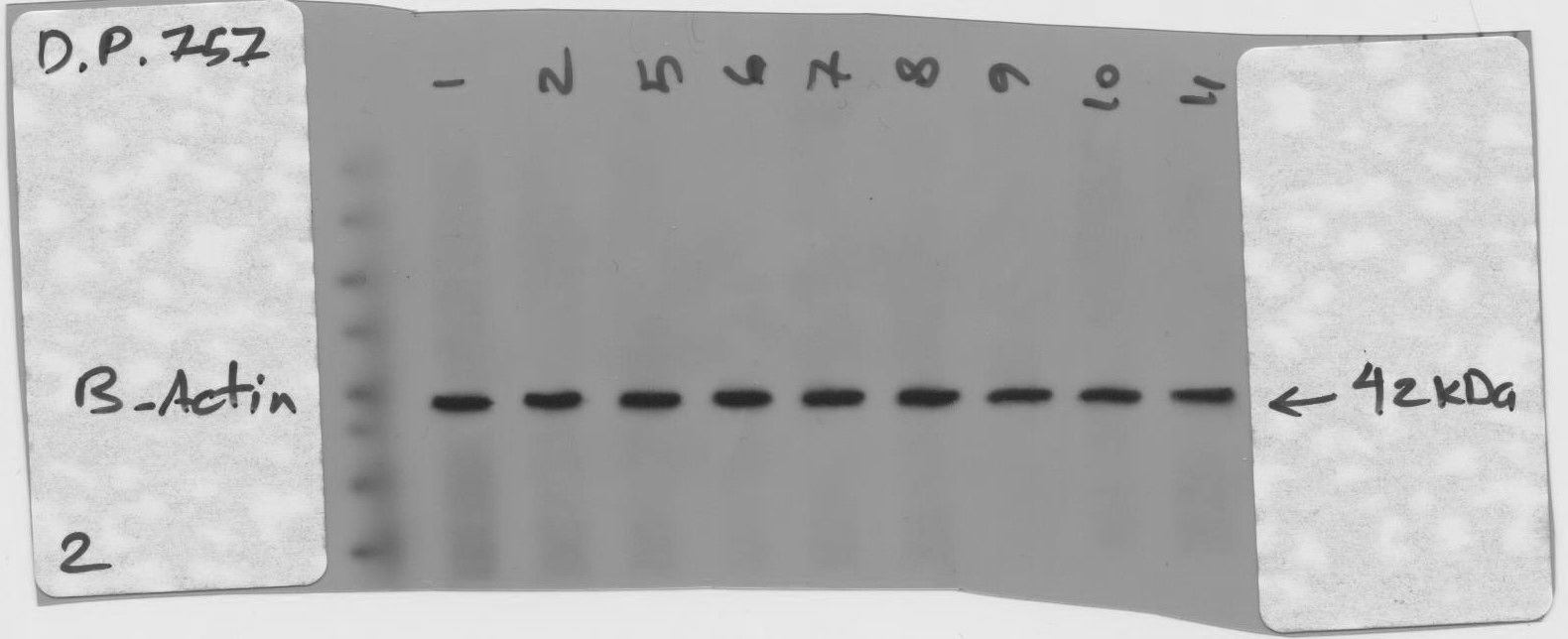
**
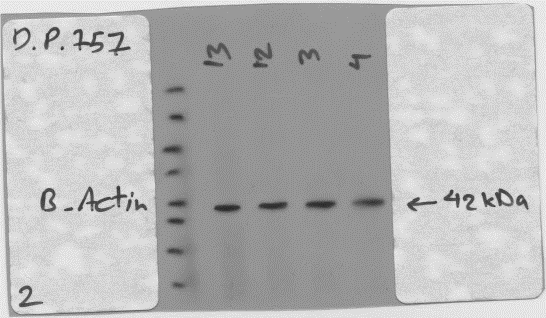
**


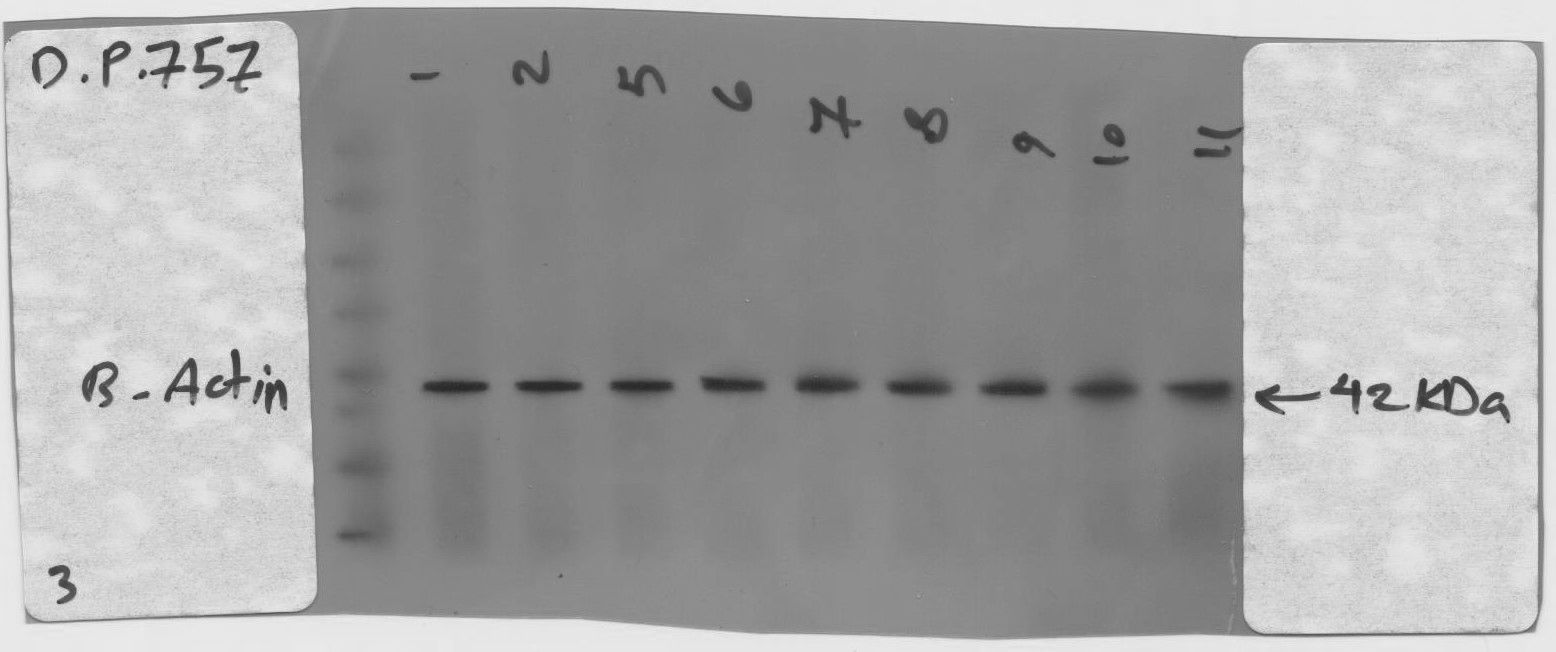


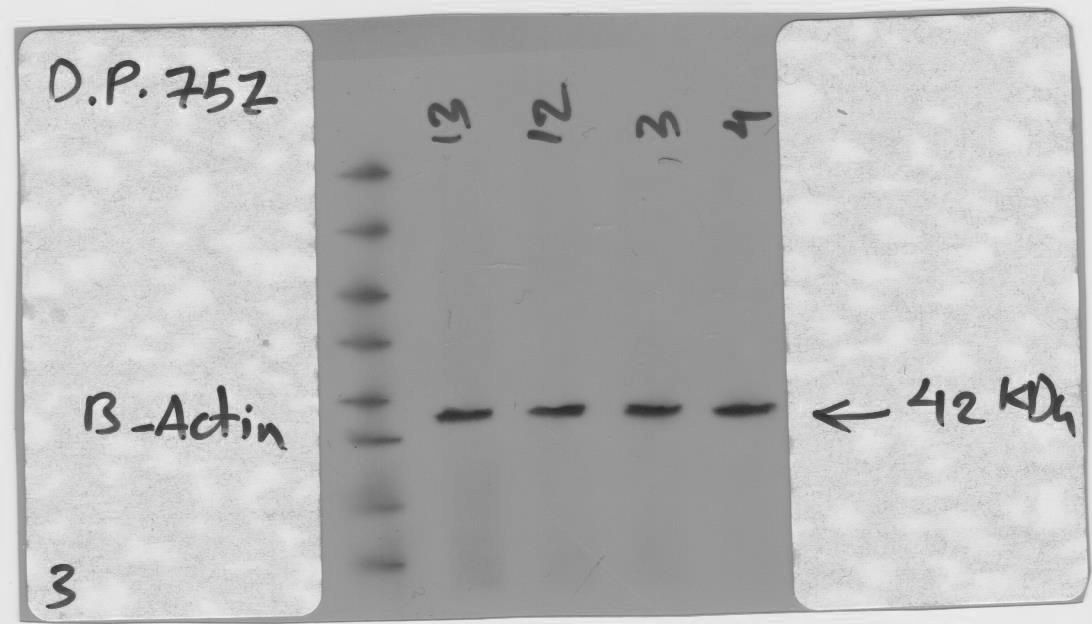


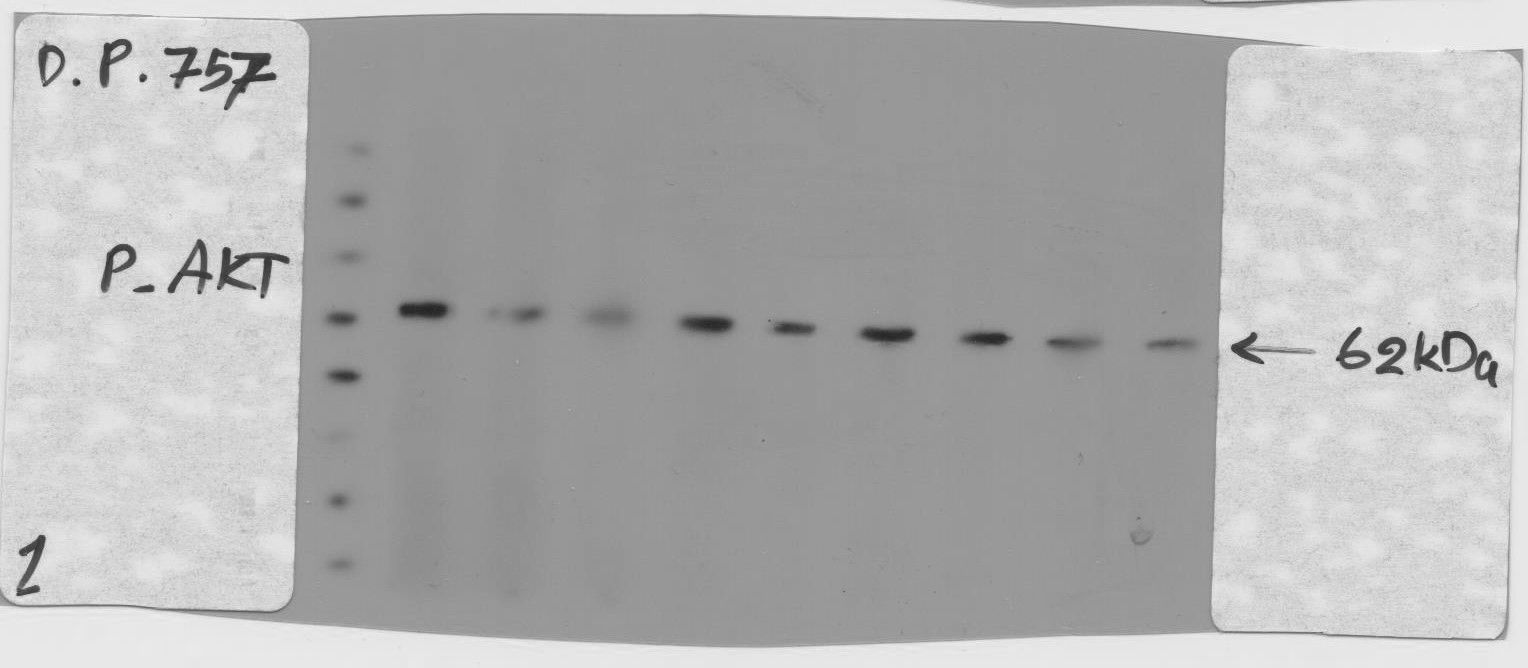

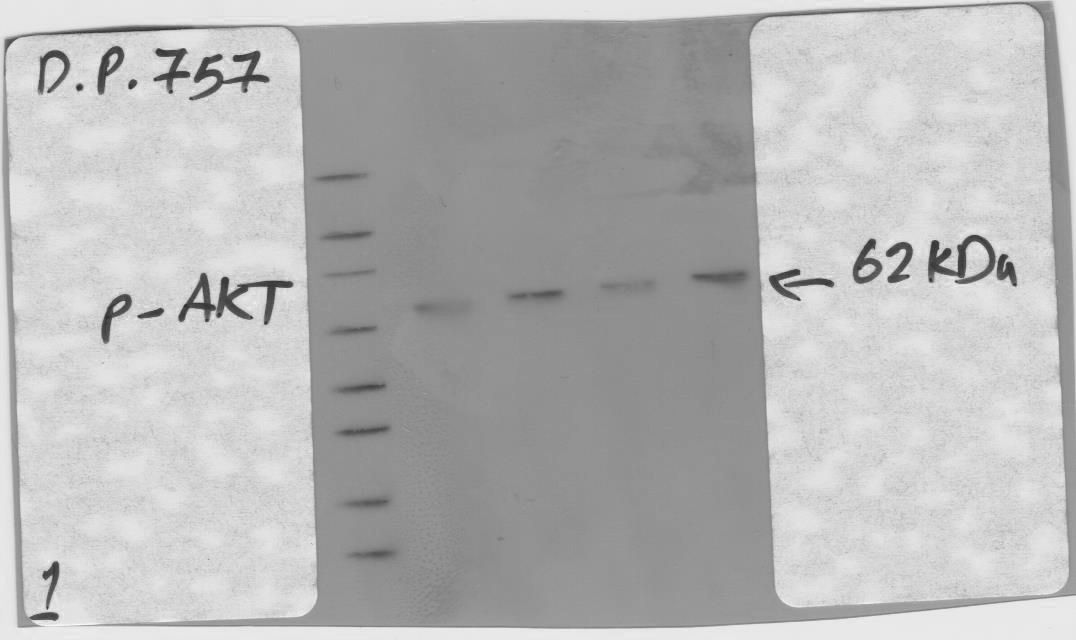


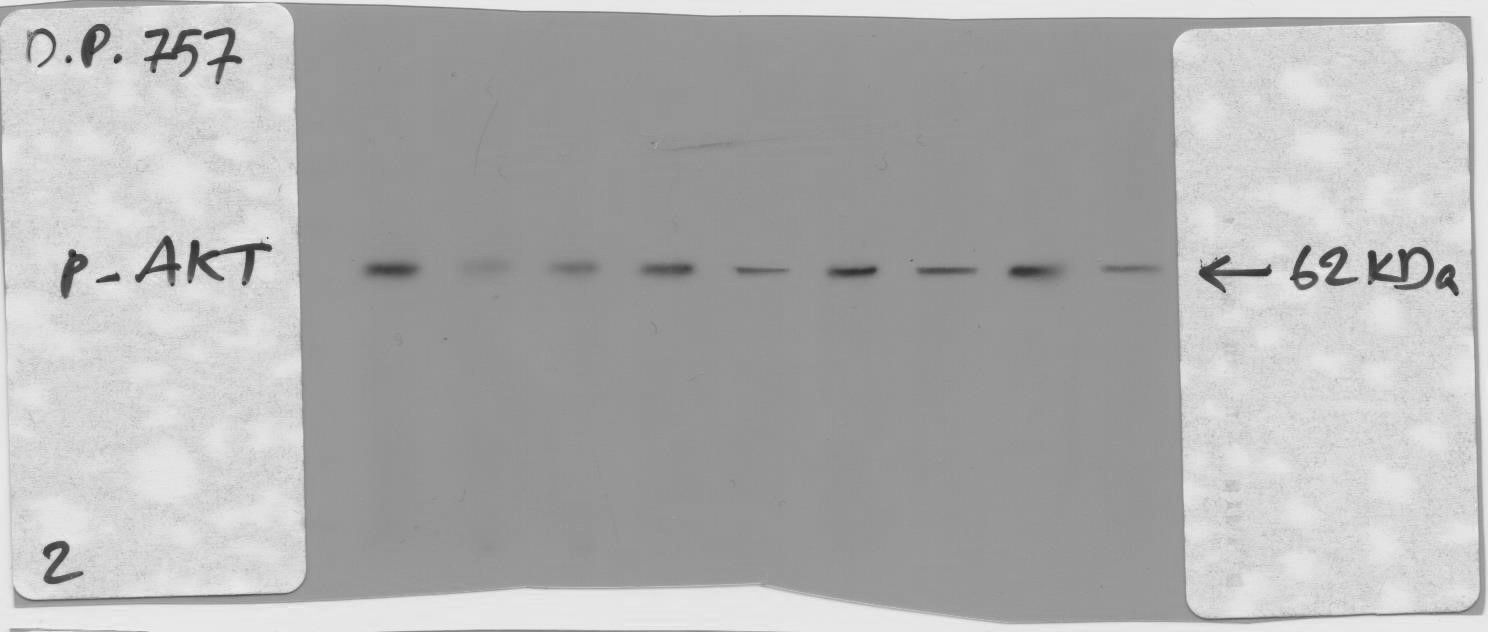

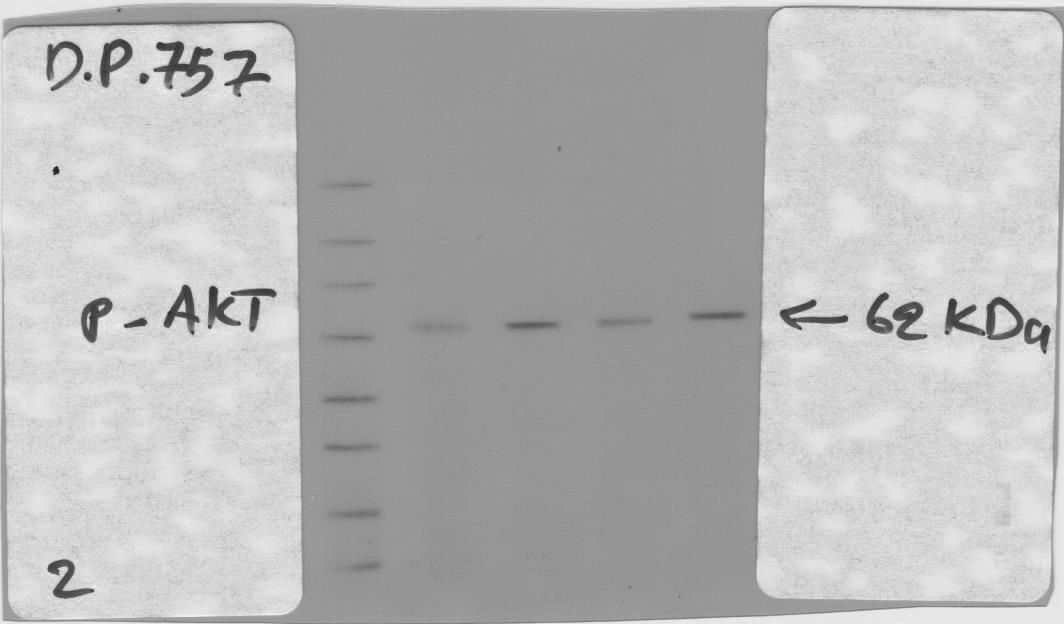


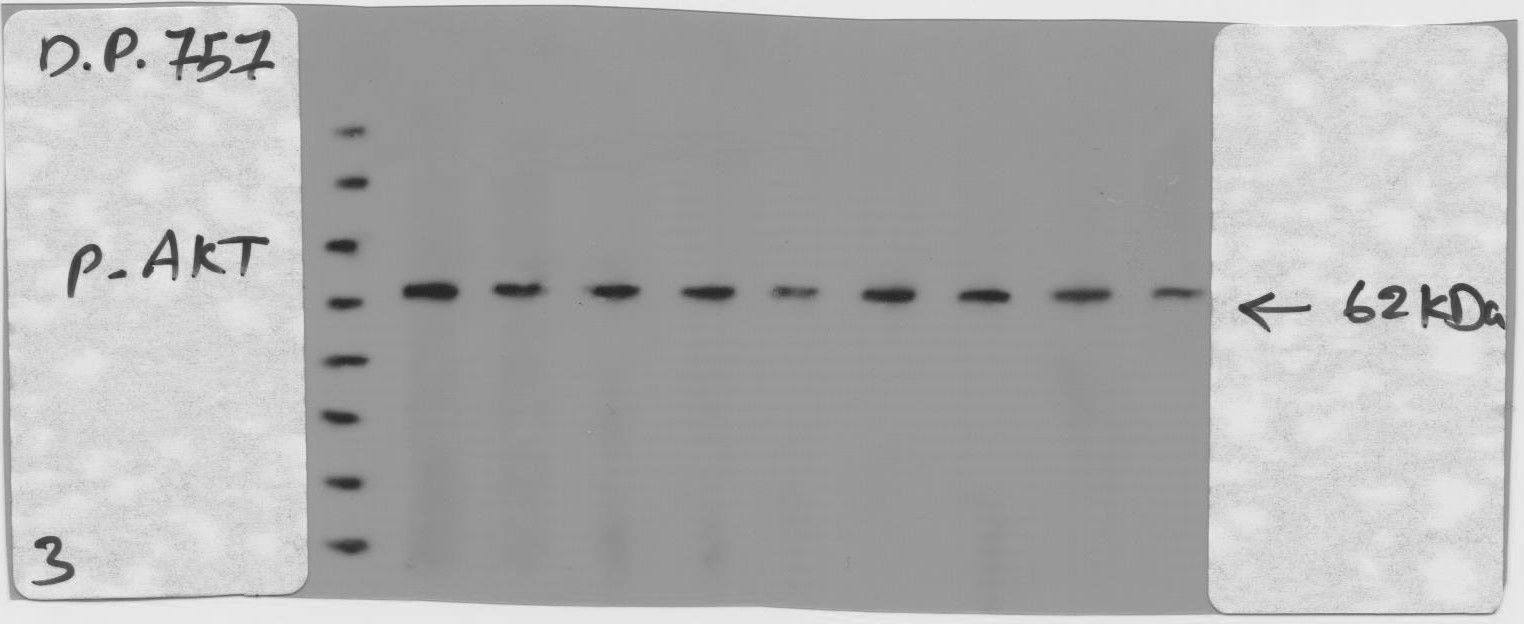

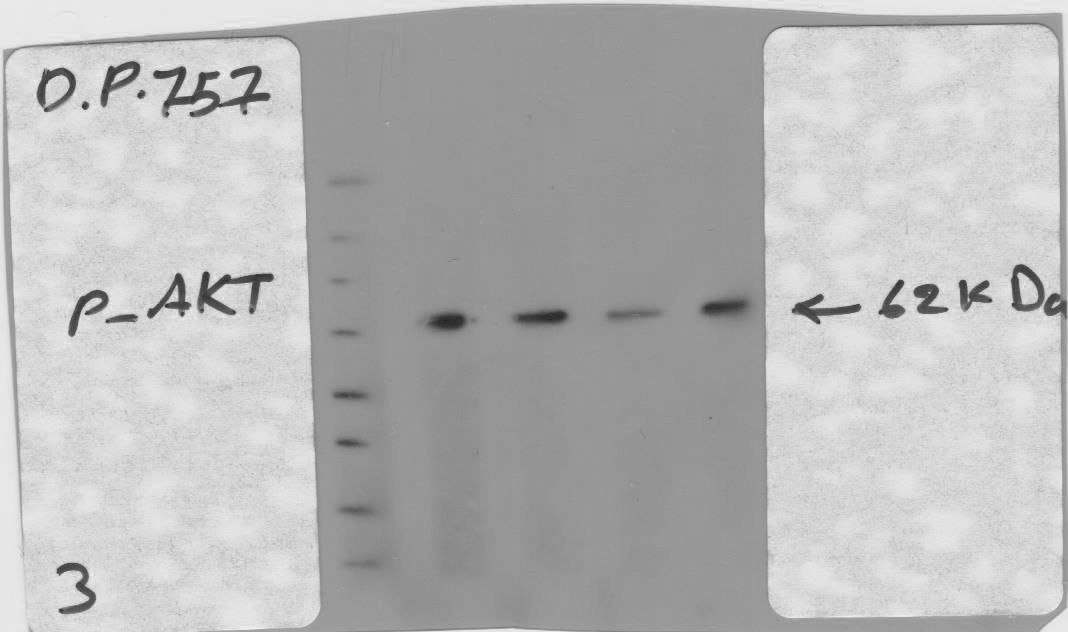


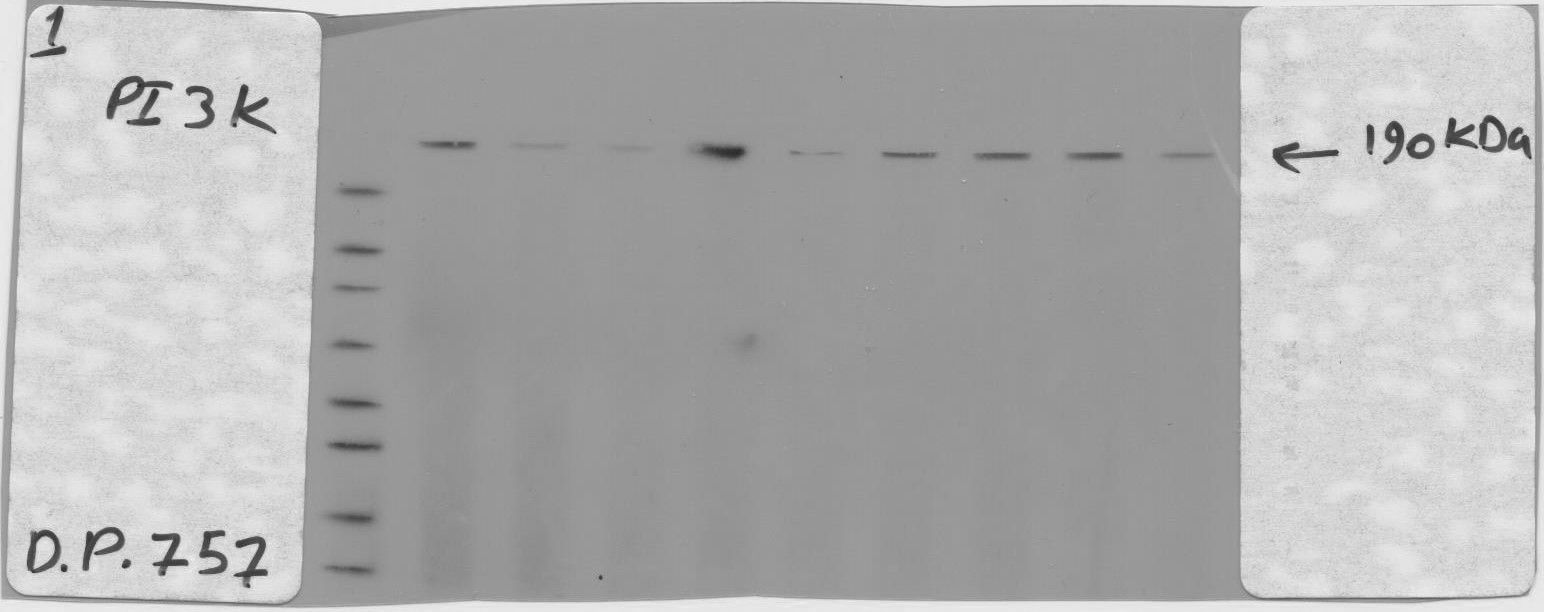

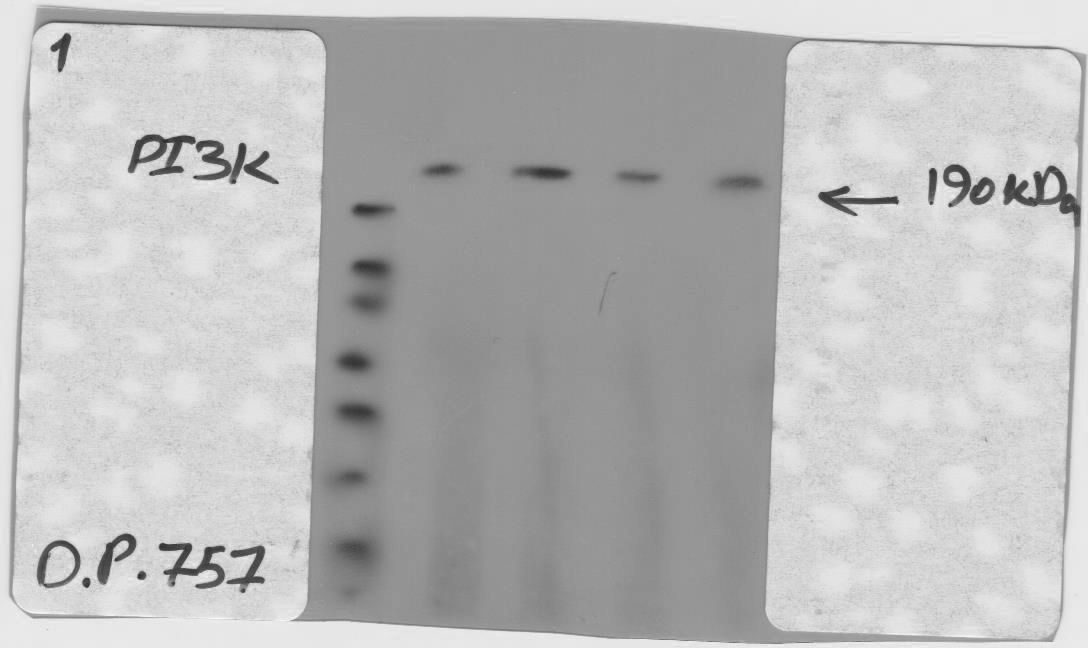


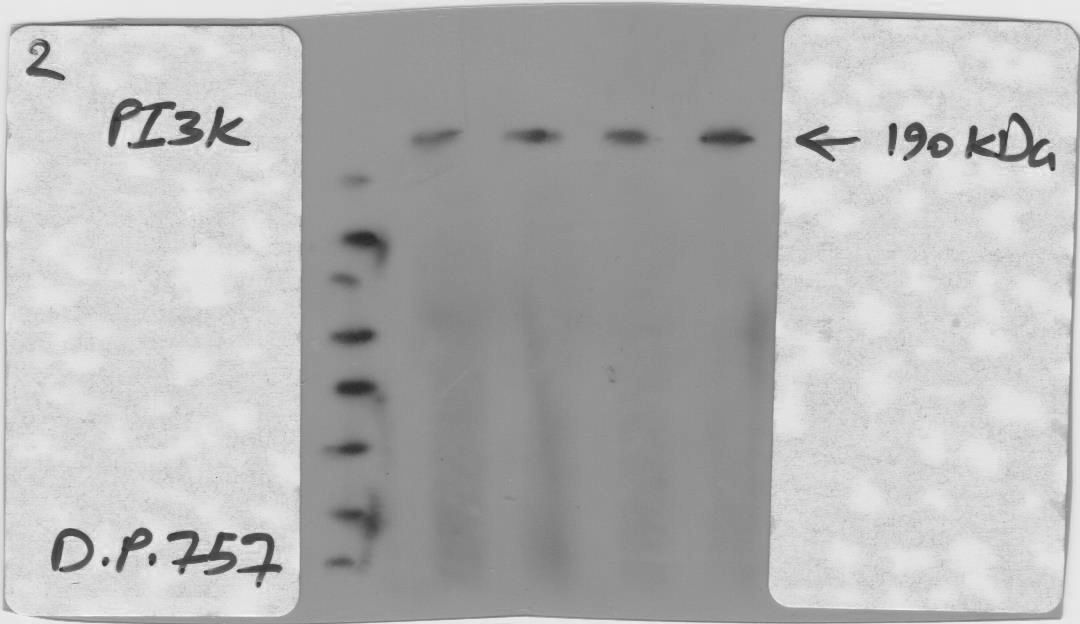

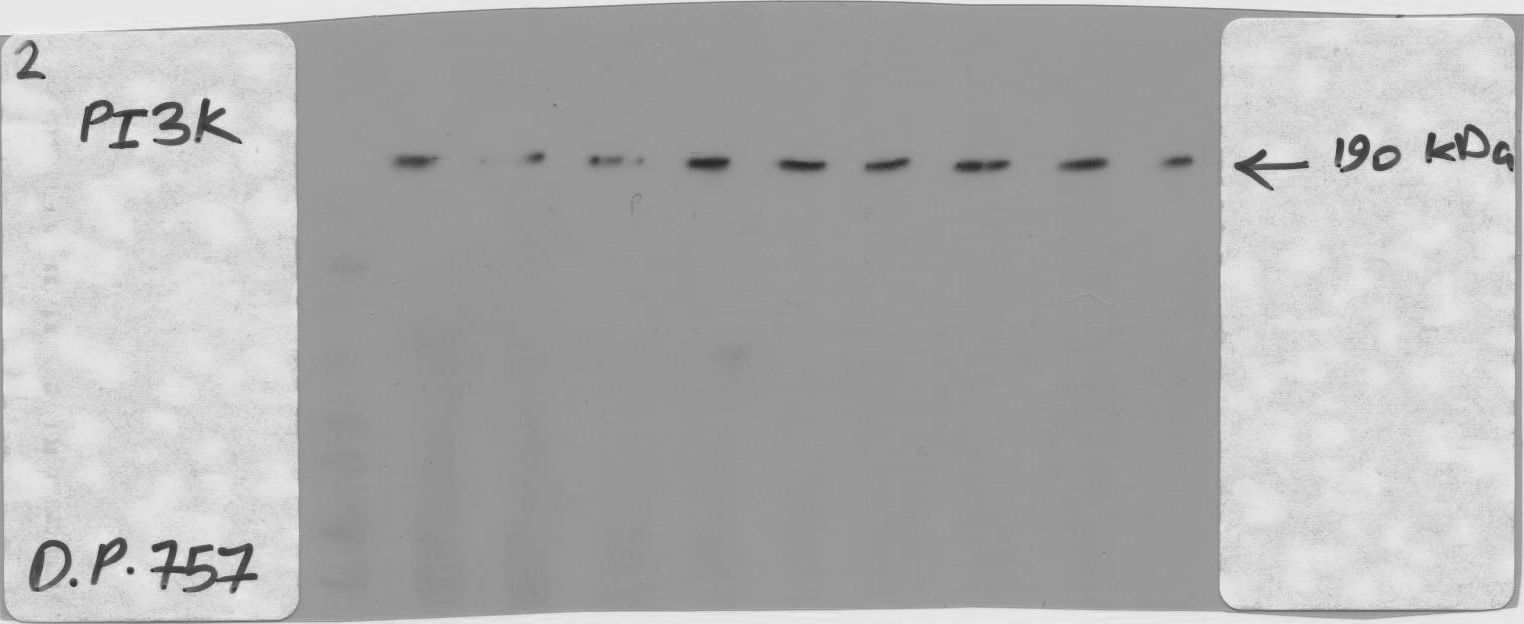

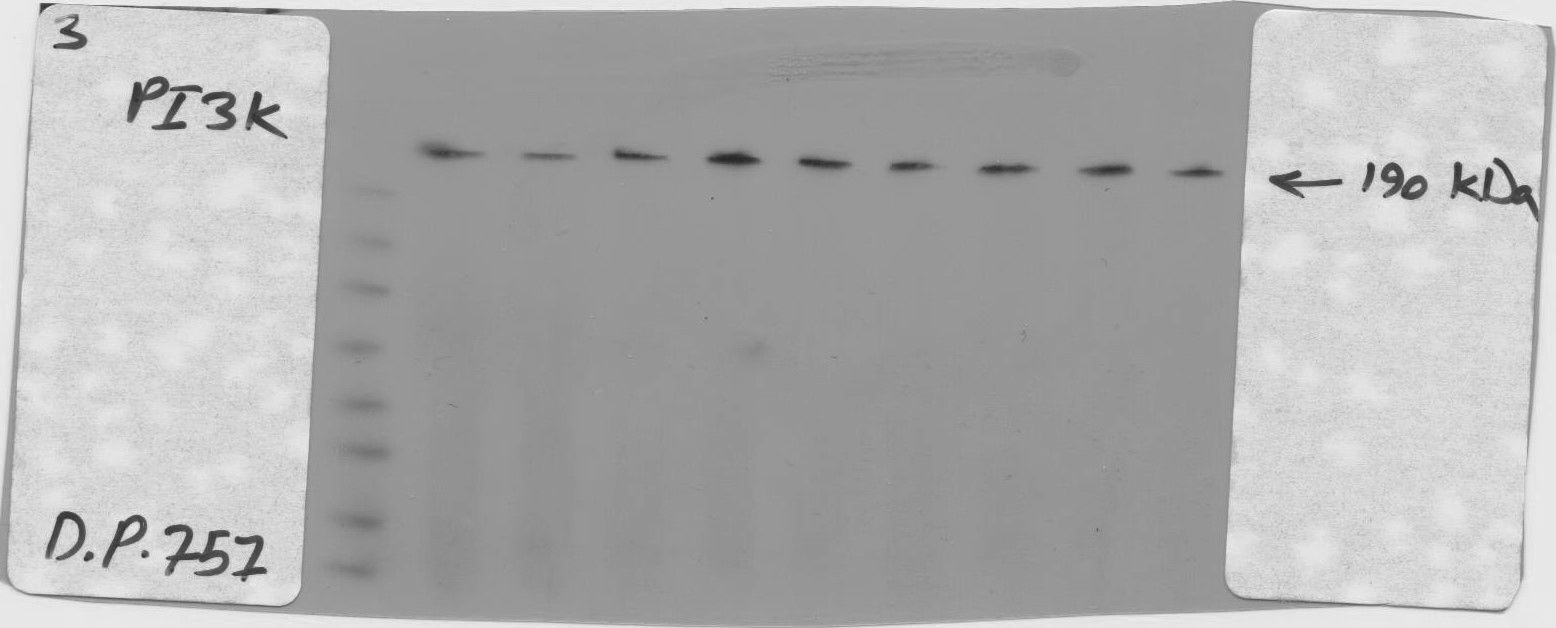
**
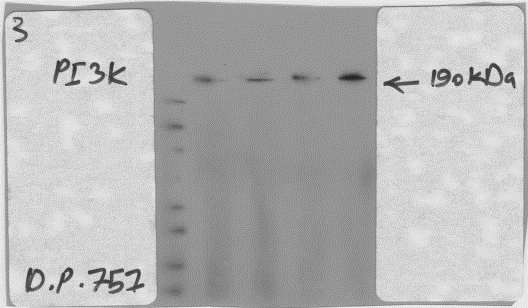
**
